# Exclusion of a major role for the *PTEN* tumour-suppressor gene in breast carcinomas

**DOI:** 10.1038/sj.bjc.6690121

**Published:** 1999-02

**Authors:** D Freihoff, A Kempe, B Beste, B Wappenschmidt, E Kreyer, Y Hayashi, A Meindl, D Krebs, O D Wiestler, A von Deimling, R K Schmutzler

**Affiliations:** Department of Obstetrics and Gynecology, University of Bonn Medical Center, Bonn, D-53105, Germany; Department of Neuropathology, University of Bonn Medical Center, Bonn, D-53105, Germany; Department of Medical Genetics, Ludwig Maximilian University, Munich, D-80336, Germany

**Keywords:** *PTEN/MMAC1/TEP1*, breast cancer, mutations, LOH10q, tumour-suppressor gene

## Abstract

*PTEN* is a novel tumour-suppressor gene located on chromosomal band 10q23.3. This region displays frequent loss of heterozygosity (LOH) in a variety of human neoplasms including breast carcinomas. The detection of *PTEN* mutations in Cowden disease and in breast carcinoma cell lines suggests that *PTEN* may be involved in mammary carcinogenesis. We here report a mutational analysis of tumour specimens from 103 primary breast carcinomas and constitutive DNA from 25 breast cancer families. The entire coding region of *PTEN* was screened by single-strand conformation polymorphism (SSCP) analysis and direct sequencing using intron-based primers. No germline mutations could be identified in the breast cancer families and only one sporadic carcinoma carried a *PTEN* mutation at one allele. In addition, all sporadic tumours were analysed for homozygous deletions by differential polymerase chain reaction (PCR) and for allelic loss using the microsatellite markers *D10S215*, *D10S564* and *D10S573*. No homozygous deletions were detected and only 10 out of 94 informative tumours showed allelic loss in the *PTEN* region. These results suggest that *PTEN* does not play a major role in breast cancer formation. *1999 Cancer Research Campaign*


*British Journal of Cancer* (1999) **79**(5/6), 754–758
© 1999 Cancer Research Campaign
Article no. bjoc.1998.0121

### Exclusion of a major role for the *PTEN* tumour-suppressor gene in breast carcinomas

**D Freihoff[1], A Kempe[1], B Beste[1], B Wappenschmidt[1], E Kreyer[1], Y Hayashi[2], A Meindl[3], D Krebs[1], OD Wiestler[2], A von Deimling[2] and RK Schmutzler[1,2]**

[1]Department of Obstetrics and Gynecology, University of Bonn Medical Center, D-53105 Bonn, Germany; [2]Department of Neuropathology, University of Bonn Medical Center, D-53105 Bonn, Germany; [3]Department of Medical Genetics, Ludwig Maximilian University, D-80336 Munich, Germany

**Summary** *PTEN* is a novel tumour-suppressor gene located on chromosomal band 10q23.3. This region displays frequent loss of heterozygosity (LOH) in a variety of human neoplasms including breast carcinomas. The detection of *PTEN* mutations in Cowden disease and in breast carcinoma cell lines suggests that *PTEN* may be involved in mammary carcinogenesis. We here report a mutational analysis of tumour specimens from 103 primary breast carcinomas and constitutive DNA from 25 breast cancer families. The entire coding region of *PTEN* was screened by single-strand conformation polymorphism (SSCP) analysis and direct sequencing using intron-based primers. No germline mutations could be identified in the breast cancer families and only one sporadic carcinoma carried a *PTEN* mutation at one allele. In addition, all sporadic tumours were analysed for homozygous deletions by differential polymerase chain reaction (PCR) and for allelic loss using the microsatellite markers *D10S215*, *D10S564* and *D10S573*. No homozygous deletions were detected and only 10 out of 94 informative tumours showed allelic loss in the *PTEN* region. These results suggest that *PTEN* does not play a major role in breast cancer formation.

**Keywords:** *PTEN/MMAC1/TEP1*; breast cancer; mutations; LOH10q; tumour-suppressor gene

Breast carcinoma represents the most common malignancy of women in Western countries. Despite its prevalence, the molecular mechanisms of breast cancer formation and progression are still poorly understood. Molecular studies suggest that tumour-suppressor genes involved in hereditary tumour formation may also be altered in their sporadic counterparts (Fearon, 1997). Five per cent of all patients with breast carcinomas report a family history and the majority of these familial cases have been associated with germline mutations of the *BRCA1* or *BRCA2* tumour-suppressor genes (Miki et al, 1994; Wooster et al, 1995). However, the *BRCA1* and *BRCA2* genes are not usually altered in sporadic breast carcinomas (Lancaster et al, 1996; Miki et al, 1996), although loss of heterozygosity (LOH) in the *BRCA1* and *BRCA2* on chromosomal arms 17q and 13q is frequently observed (Schmutzler et al, 1997).

Recently, the *PTEN*/*MMAC1*/*TEP1* tumour-suppressor gene has been identified on chromosomal band 10q23.3 (Li and Sun, 1997; Li et al, 1997; Steck et al, 1997). The product of this gene harbours a tyrosine phosphatase domain which shares high sequence homology with the cytoskeleton proteins tensin and auxilin. Mutations of *PTEN* were observed in a variety of tumours including breast carcinoma cell lines and primary invasive breast carcinomas (Li et al, 1997; Steck et al, 1997). These mutations included homozygous deletions and frameshift or nonsense mutations. Moreover, loss of heterozygosity affecting 10q23.3 was detected in as many as 50% of primary breast carcinomas. Germline mutations of *PTEN* have been identified in Cowden disease, a rare autosomal dominant cancer syndrome characterized by malignancies of the breast, thyroid and brain (Liaw et al, 1997). These observations point to *PTEN* as an interesting candidate for a tumour-suppressor gene associated with breast cancer.

Recent studies on sporadic tumours demonstrated that mutations in the *PTEN* gene are frequent events in glioblastomas, malignant melanomas and endometrial carcinomas of the endometrioid type (Guldberg et al, 1997; Kong et al, 1997; Rasheed et al, 1997; Tashiro et al, 1997; Wang et al, 1997). In endometrial carcinomas, *PTEN* mutations were predominantly found in tumours with microsatellite instability (Kong et al, 1997; Tashiro et al, 1997). It remains to be shown whether the association of *PTEN* mutations and microsatellite instability is of biological significance. In a series of 54 sporadic breast carcinomas, two deletions resulting in truncated proteins and various missense mutations of unknown significance have been reported (Rhei et al, 1997). To further elucidate the potential role of *PTEN* in breast carcinomas, we analysed 103 sporadic breast carcinomas for mutations, homozygous deletions and loss of heterozygosity, and 25 families with hereditary breast cancer for constitutive mutations in the *PTEN* gene.

### MATERIALS AND METHODS

### Tumour specimens from sporadic breast carcinomas and blood samples from patients with hereditary breast carcinomas

Breast cancer families were recruited at the University Hospital Bonn and the Department of Medical Genetics, University of Munich, Germany. Our series comprised 25 families fulfilling the following criteria: (1) at least two affected female relatives with breast or ovarian cancer and at least one patient with age at manifestation of less than 50 years; (2) one affected female with two

*Received 20 January 1998*
*Revised 29 May 1998*
*Accepted 2 June 1998*

*Correspondence to:* RK Schmutzler, Universitäts-Frauenklinik, Sigmund-Freud Str. 25, D-53105 Bonn, Germany cancers, either bilateral breast cancer or breast and ovarian cancer; (3) one affected female with an age of onset for breast cancer under 35 years. Male breast cancer did not occur in these families. Most of the families were high-risk families with at least three breast or ovarian carcinomas (Table 1). *BRCA1* mutations were excluded in all families by complete sequencing of the coding region including exon/intron boundaries. *BRCA2* mutation analysis is currently under investigation and so far excluded by single-strand conformation polymorphism (SSCP) analysis in five families. In addition, in ten families frequent mutations in the *BRCA2* gene could be excluded by direct sequencing of exons 9, 23 and 27.

Tissue was obtained from 103 women undergoing surgery for sporadic breast carcinomas at the University Hospital Bonn, the St. Elisabeth Hospital Bonn-Bad Godesberg and the Marienhospital Bruehl, Germany. Malignant tumours were grouped according to the UICC TNM (Spiessl et al, 1990) classification. The WHO classification was used for histopathological analysis (World Health Organization, 1981). Histological grading was performed according to Bloom and Richardson (1957). The oestrogen and progesterone receptor (ER and PR) status were determined by the dextran-coated charcoal (DCC) method or by a monoclonal antibody assay (Remmele et al, 1986). Thirteen of the carcinomas represented recurrent or metastatic tumours, 88 primary carcinomas, and from two no data were available. Among the primary carcinomas, 36 corresponded to pT1 and 40 were larger tumours (pT > 1). From 12 tumours, no data were available. Forty-nine tumours were classified as pN0, 38 were characterized by lymph node infiltration, and from one tumour no staging data was available. Sixty-seven carcinomas were histopathologically classified as ductal carcinomas, nine as lobular carcinoma, two as medullary carcinoma, two as mucinous carcinomas, one as tubular carcinoma and three as ductal carcinoma in situ. From four tumours, no histological subclassification was obtained. Twenty-four carcinomas were classified as grade III, 59 tumours as grade II or I, and from five tumours grading was not available. Seventeen tumours were ER negative and 66 ER positive. PR expression was detected in 50 tumours and undetectable in 33 tumours. For five carcinomas, the hormone receptor status was not available.

Tissue samples were stored at –80°C and blood samples at –20°C until further treatment. Tumour tissue was processed by microdissection to exclude regions of normal breast tissue and only samples containing more than 90% tumour cells were investigated. DNA was extracted from tissue and peripheral leucocytes using a conventional phenol–chloroform protocol.

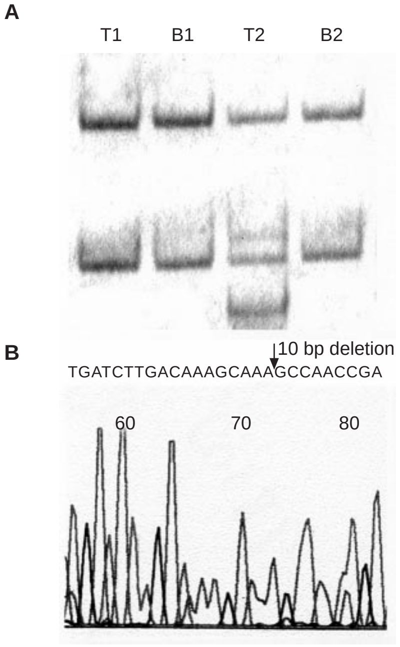

**Figure 1** Somatic *PTEN* mutation in one breast carcinoma. (**A**) The silver-stained SSCP gel shows an aberrant migration pattern of the amplified tumour DNA sample T2 (T, tumour) in comparison with the corresponding blood sample B2 (B, blood). (**B**) Sequence analysis of the tumour DNA revealed a 10-bp deletion in exon 8 leading to a premature stop codon in exon 9

### SSCP analysis and DNA sequencing

For a mutational analysis of the *PTEN* gene, intronic and overlapping exonic primers were used for amplification (Duerr et al, 1998). The primers cover the entire coding sequence as well as the exon/intron boundaries of the *PTEN* gene. Polymerase chain reaction (PCR) was performed in a volume of 10 µl containing 20 ng of DNA, 50 mM potassium chloride, 10 mM tris-HCl, 200 µM of each dNTP, 0.1% gelatin, 10 pmol of each primer, 1.0–2.0 mM magnesium chloride and 0.25 U *Taq* polymerase. Initial denaturation at 94°C for 3 min was followed by 30 cycles on an automated thermal cycler (Hybaid, Omnigene, USA). Denaturation at 94°C for 30 s was followed by annealing at 50–55°C for 40 s and extension at 72°C for 40 s. A final extension step at 72°C for 10 min was added. Single-strand conformation polymorphism (SSCP) analysis was carried out on a sequencing apparatus (Pokerface II,

**Table 1** Characteristics of the families analysed for *PTEN* mutations

| | Number of cases per family | Number of families | *BRCA1* Mutation analysis | *BRCA2* Mutation analysis |
|---|---|---|---|---|
| Breast cancer only | 1* | 2 | All neg. | All neg. for f.m. |
| | 2 | 4 | All neg. | All neg. for f.m. |
| | 3 | 7 | All neg. | 3 fam. neg.<br>1 fam. neg. for f.m. |
| | ≥4 | 6 | All neg. | 2 fam. neg. |
| Breast and ovarian cancer | 3 | 1 | All neg. | All neg. for f.m. |
| | ≥4 | 5 | All neg. | 2 fam. neg. for f.m. |

Clinical characteristics of the analysed families (fam.) at risk for breast and/or ovarian cancer. *In the two families with one case of breast cancer only, ages at manifestation were 33 and 34 years. All families were negative (neg.) for *BRCA1* mutations. *BRCA2* mutations could be excluded in five families. Additionally, ten families were tested negative for frequent *BRCA2* mutations (f.m.) in the exons 9, 23 and 27.

© Cancer Research Campaign 1999

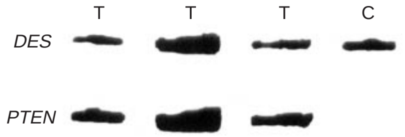

**Figure 2** Analysis of homozygous deletions in sporadic breast carcinomas. Genomic DNA samples of breast carcinomas (T) were amplified by PCR using intronic primers. DNA from the glioblastoma cell line A172 served as positive control (C). A 160-bp fragment including exon 4 of the *PTEN* gene was coamplified with a 171-bp fragment from intron 7 of the *DESMIN* gene. Bands were detected by the semiautomatic sequencer Licor 4200 and analysed with the One-Dscan-Software. Significant differences between *DESMIN* and *PTEN* signal intensity were only observed for the DNA template of the glioblastoma cell line

Hoefer, San Francisco, USA) using 10% acrylamide gels with a bisacrylamide:acrylamide ratio of 1:59 or 12% acrylamide gels with a bisacrylamide:acrylamide ratio of 1:29 and 1:79, electrophoresis at 5–10 W and variable temperatures for 14 h. Silver staining of the gels was performed as previously described (von Deimling et al, 1993; Bender et al, 1994). Aberrantly migrating SSCP bands were excised and the DNA was extracted. After reamplification with the same set of primers, the PCR products were sequenced on a semiautomated sequencer (Applied Biosystems, model 310, Foster City, USA) using a *Taq* cycle sequencing kit (Abi Prism) dye terminator cycle sequencing ready reaction kit, Perkin Elmer, Alameda, USA).

Under the same conditions used here, we previously detected 32 mutations in brain tumours (Duerr et al, 1998). These mutations included deletions, insertions, missense and nonsense mutations in exons 1–8.

### Analysis for homozygous deletions

A PCR assay for the detection of homozygous deletions has been described previously (Hayashi et al, 1997). In brief, a 160-bp fragment including exon 4 of the *PTEN* gene was coamplified with a 171-bp fragment from intron 7 of the *DESMIN* (*DES*, chromosome 2) gene as a control (Duerr et al, 1998). One primer of each pair was labelled with infrared dye 41 (MWG-Biotech, Ebersberg, Germany) at the 5′ end. Differential PCR was performed in a final volume of 10 µl containing 10 ng DNA, 50 mM potassium chloride, 1.5 mM magnesium chloride, 10 mM tris-HCl, pH 8.3, 200 µM of each dNTP, 0.1% gelatin, 5 pmol of each primer, and 0.25 U *Taq* polymerase (Gibco-BRL). Initial denaturation at 94°C for 3 min, was followed by 28 cycles on an automated thermocycler (Hybaid, Omnigene). These included denaturation at 94°C for 40 s, annealing at 56°C for 55 s and extension at 72°C for 55 s. A final extension step of 10 min at 72°C was used. Fluorescent PCR products were separated on a 6% polyacrylamide gel and analysed on a semiautomated DNA sequencer (Licor, Lincoln, NE, USA). Quantitative analysis of the signal intensity was carried out with the One-Dscan program (Scanalytics). To determine the *PTEN*/*DES* ratios in normal DNA samples, leucocyte DNA from 40 healthy controls and patients was analysed. *PTEN:DES* ratios ranged from 1.5 to 3.2, averaging 2.2 with a standard deviation (s.d.) of 0.41. *PTEN:DES* ratios lower than mean minus 3 s.d., i.e. <0.97, were considered as indicative of *PTEN* deletions. DNA of the glioblastoma cell line A172 lacking the Cowden critical region was used as a positive control (Li et al, 1997).

### Detection of allelic imbalance

Three microsatellite markers *D10S573*, *D10S215* and *D10S564* located on chromosomal band 10q23.1–23.3 were used for the detection of allelic loss in the *PTEN* genomic region. With these same markers, linkage analysis provided highest lod scores in Cowden disease families and the Cowden locus was mapped between markers *D10S215* and *D10S564* (Nelen et al, 1997). Eighty of these tumours were also analysed for allelic loss in the regions of the *BRCA1*, *BRCA2* and *TP53* genes and 16q24 using the microsatellite markers *D17S855*, *D13S267*, *TP53* and *D16S539*.

Two dinucleotide repeats (*D2S136* and *D5S346*) and two mononucleotide repeats (*BAT25* and *BAT26*) previously identified as frequent targets for microsatellite instability in hereditary non-polyposis colon carcinoma (HNPCC) were examined in a panel of 11 carcinomas (Bocker et al, 1997).

Genomic DNA (100 ng) from tumours and corresponding leucocytes was used as template. PCR was performed in a volume of 10 µl containing 20 ng of DNA, 50 mM potassium chloride, 10 mM tris-HCl, 200 µM of each dNTP, 0.1% gelatin, 10 pmol of each primer, 1.5 mM magnesium chloride and 0.025 U *Taq* polymerase. Initial denaturation at 94°C for 3 min was followed by 32 cycles on an automated thermal cycler (Bio-med 623, Theres, Germany). These included denaturation at 94°C for 50 s, annealing at 53°C (*D10S215*), 57°C (*D10S564*) and 59°C (*D10S573*) for 50 s and extension at 72°C for 40 s. A final extension step at 72°C for 10 min was added. Loss of heterozygosity analysis was performed on a sequencing apparatus (Pokerface II, Hoefer) using denaturating 8% acrylamide gels with a bisacrylamide:acrylamide ratio of 1:19 and electrophoresis at 75 W for 2.5 h. Silver staining of the gels was carried out as previously described (von Deimling et al, 1993; Bender et al, 1994).

### RESULTS

### Mutation detection by SSCP and direct sequencing

The index patients of the 25 breast cancer families were examined for germline mutations in *PTEN*. *BRCA1* mutations had previously been excluded. Five families were negative for *BRCA2* mutations and an additional ten families were negative for frequent mutations in exons 9, 23 and 27. No abberant migration patterns of the *PTEN* SSCP fragments could be detected in any of these patients.

One of the 103 sporadic tumours showed an altered migration pattern. Sequence analysis revealed a 10-bp deletion within exon 8 resulting in a truncated protein with a stop signal at codon 343 in exon 9. This mutation was proven to be of somatic origin by analysis of constitutional DNA (Figure 1). No LOH could be observed in this tumour sample for the other allele. The tumour occurred in a 67-year-old woman and was histopathologically classified as an invasive ductal carcinoma, pT1c, pN0, pM0, WHO grade III, oestrogen and progesterone positive.

### Homozygous deletions

Our tumour samples were also screened for homozygous deletions. Carcinoma samples with *PTEN:DES* ratios lower than mean minus 3 s.d., i.e. <0.97, were considered to have *PTEN* deletions in order to introduce a correction factor for the contamination of breast carcinoma tissue with stromal cells. Applying this calculation, none of the tumours showed evidence for a homozygous deletion of the *PTEN* gene (Figure 2).

 © Cancer Research Campaign 1999

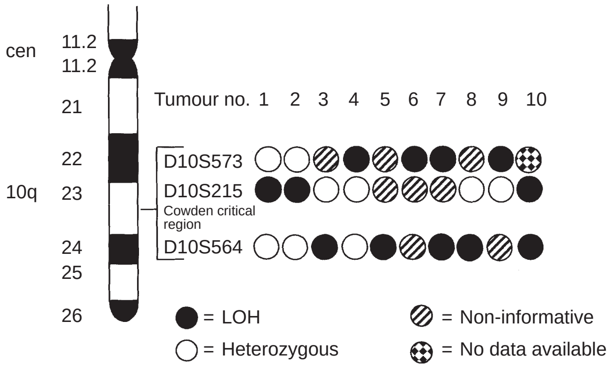

**Figure 3** LOH analysis of markers closely spaced to the Cowden critical region. In 10 of 92 informative carcinomas, LOH was observed in at least one marker, but in only two tumours (no. 7 and no. 10) the Cowden critical region was flanked by LOH

### Loss of heterozygosity

Analysis of three microsatellite markers in 92 informative carcinomas revealed ten tumours with LOH10q in at least one locus. However, only two carcinomas exhibited LOH including the entire Cowden critical region (Figure 3).

LOH analysis in other chromosomal regions revealed frequencies of 20% for the marker *D17S855* (16 out of 79 informative tumours), 20% for the marker *D13S267* (13 out of 68), 41% for the marker *TP53* (13 out of 32) and 60% for the marker *D16S539* (30 out of 51).

### Microsatellite instability

To examine a potential association of *PTEN* mutations with microsatellite instability, four microsatellite markers located on different chromosomes were analysed in a panel of 11 carcinomas including ten carcinomas without *PTEN* mutations and the one tumour with a *PTEN* mutation. No evidence for microsatellite instability was observed with any of these markers.

### DISCUSSION

Recent reports on mutations of the *PTEN* tumour-suppressor gene in Cowden disease and in a variety of different tumour entities have raised the possibility of an involvement of *PTEN* in breast carcinogenesis (Li et al, 1997; Liaw et al, 1997; Steck et al, 1997). We here present data of a *PTEN* mutational analysis in 103 sporadic breast carcinomas and 25 families afflicted with hereditary breast and ovarian cancer. The results strongly indicate that alterations of the *PTEN* gene are a rare event in breast carcinomas.

Analysis of *PTEN* in Cowden syndrome and early onset familial breast cancer also failed to detect germline mutations in this subset of breast cancer families (Tsou et al, 1997). Moreover, the authors of a recent report failed to detect any germline mutation in the *PTEN* gene in a set of 136 breast cancer families in which alterations of *BRCA1*, *BRCA2*, *TP53* and *ATM* were previously excluded (Chen et al, 1998). Our analysis comprised families with breast and/or ovarian cancer not restricted to early onset of the disease. The absence of germline mutations in both studies implies that *PTEN* does not contribute significantly to the formation of hereditary breast and ovarian cancer. Rhei et al (1997) described a somatic 2-bp deletion and a 4-bp germline deletion in a series of 54 unselected primary breast cancers. Subsequent clinical examination attributed the germline deletion to a family history of Cowden syndrome. Ueda et al (1998) detected only one missense mutation in a series of 69 primary breast cancers. These data are in accordance with our results demonstrating that *PTEN* mutations are an infrequent event in primary sporadic breast cancer. Moreover, in contrast to initial results on breast cancer cell lines (Li et al, 1997), our findings indicate that homozygous deletions are uncommon in primary breast carcinomas.

In addition to the two mutations, Rhei et al (1997) identified several missense variants that occurred in most of the tumour samples. Recently, a highly conserved and processed *PTEN* pseudogene has been found on chromosomal band 9p21 (Bostroem et al, 1998; Dahia et al, 1998). This gene shares 98% sequence homology with the coding region of functional *PTEN*. Analysis of cDNA from a glioblastoma cell line with *PTEN* deletion suggests that the pseudogene is transcriptionally inactive. Nine of the 11 variants described by Rhei et al (1997) align with the pseudogene sequence. As this analysis was performed with a cDNA template, any of the mRNA variants may well represent a DNA pseudogene contamination of the mRNA samples. Alternatively, the pseudogene may be expressed in breast carcinoma. However, in our analysis of genomic DNA from 103 primary tumours, we could not detect any sequence variation.

The association of *PTEN* mutations in microsatellite instability-positive endometrial carcinomas (Tashiro et al, 1997) raised the possibility that *PTEN* may be associated with microsatellite instability. This property is also supported by the detection of microsatellite instability on chromosomal band 10q11–qter in primary breast carcinomas (Sourvinos et al, 1997). It was, therefore, of interest to analyse these tumours for microsatellite instability. However, our analysis with four microsatellite markers that represent frequent targets of replication error did not reveal microsatellite instability neither in the tumour sample exhibiting a *PTEN* mutation nor in ten breast carcinomas without mutant *PTEN*.

In contrast to the initial reports of high allelic loss rates in the *PTEN* region on chromosome 10q in 50% and 34% of primary breast carcinomas respectively (Li et al, 1997; Steck et al, 1997), our analysis revealed LOH10q23 in less than 10% of the tumours. No allelic loss was detected in the carcinoma carrying a *PTEN* mutation. This is in agreement with the observation of Kerangueven et al (1997), who also failed to identify a significant incidence of LOH10q in a panel of 115 breast carcinomas. However, the investigators of a recent report (Singh et al, 1998) could detect loss of heterozygosity close to the *PTEN* region in 9 out of 22 breast carcinomas. LOH10q23 in these tumour samples was associated with high-grade tumours. This is in accordance with Rasheed et al (1997), who suggested that in gliomas *PTEN* mutations are restricted to high-grade tumours. The low frequency of LOH10q23 in our tumour panel may be explained by the predominance of early stage tumours with a favourable prognosis. This is supported by the detection of significant LOH frequencies of 20–60% in other chromosomal regions frequently altered in early stage breast carcinomas.

In addition, we could not detect homozygous *PTEN* deletions in our primary breast carcinoma samples as reported by other groups (Li et al, 1997; Steck et al, 1997). To detect focal homozygous deletion, we have chosen to amplify exon 4 because the minimal region of loss occurred between exons 2 and 5 in tumour cell lines (Steck et al, 1997). It is, therefore, unlikely that we have missed a significant portion of homozygous deletions.

© Cancer Research Campaign 1999

In summary, our findings suggest that *PTEN* does not play a major role in the pathogenesis of sporadic and hereditary breast cancer. It remains to be studied whether another, yet unidentified, tumour-suppressor gene on chromosome 10q participates in mammary carcinogenesis.

### ACKNOWLEDGEMENT

We thank our collegues Dr K Jäger, Dr P Citoler, Dr N Wernert for supplying tumour material and histopathological data. The excellent technical assistance by Eva-Maria Duerr and Birgit Meyer-Puttlitz is gratefully acknowledged. This study was supported by the Deutsche Forschungsgemeinschaft (Schm 800/2–3, 800/2–4), Deutsche Krebshilfe, BONFOR programme and the Schäfersnolte Foundation.

### REFERENCES

Bender B, Wiestler OD and Von Deimling A (1994) A device for processing large acrylamide gels. *Biotechniques* **16**: 204–206

Bloom HJ and Richardson WW (1957) Histological grading and prognosis in breast cancer. A study of 1049 cases of which 359 have been followed for 15 years. *Br J Cancer* **11**: 359–377

Bocker T, Diermann J, Friedl W, Gebert J, Holinski-Feder E, Karner-Hanusch J, von Knebel-Doeberitz M, Koelble K, Moeslein G, Schackert HK, Wirtz HC, Fishel R and Ruschoff J (1997) Microsatellite instability analysis: a multicenter study for reliability and quality control. *Cancer Res* **57**: 4739–4743

Bostroem J, Cobbers JMJL, Wolter M, Tabatabai G, Weber RG, Lichter P, Collins VP and Reifenberger G (1998) Mutation of the *PTEN* (MMAC1) tumor suppressor gene in a subset of glioblastomas but not in meningiomas with loss of chromosome 10q. *Cancer Res* **58**: 29–33

Chen J, Lindblom P and Lindblom A (1998) A study of the *PTEN/MMAC1* gene in 136 breast cancer families. *Hum Genet* **102**: 124–125

Dahia LM, FitzGerald MG, Zhang X, Marsh DJ, Zheng Z, Pietsch T, von Deimling A, Haluska FG, Haber DA and Eng C (1998) A highly conserved processed *PTEN* pseudogene is located on chromosome band 9p21. *Oncogene* **16**: 2403–2406

Duerr EM, Rollbrocker B, Hayashi Y, Peters N, Meyer-Putlitz B, Louis DN, Schramm J, Wiestler OD, Parsons R, Eng C and von Deimling A (1998) *PTEN* mutations in gliomas and glioneuronal tumors. *Oncogene* **15**: 2254–2259

Fearon ER (1997) Human cancer syndromes: clues to the origin and nature of cancer. *Science* **278**: 1043–1050

Guldberg P, thor Straten P, Birck A, Ahrenkiel V, Kirkin AF and Zeuthen J (1997) Disruption of the *MMAC1/PTEN* gene by deletion or mutation is a frequent event in malignant melanoma. *Cancer Res* **57**: 3660–3663

Hayashi Y, Ueki K, Waha A, Wiestler OD, Louis DN and von Deimling A (1997) Association of EGFR gene amplification and CDKN2 (p16/MTS1) gene deletion in glioblastoma multiforme. *Brain Pathol* **7**: 871–875

Kerangueven F, Eisinger F, Noguchi T, Allione F, Wargniez V, Eng C, Padberg G, Theillet C, Jacquemier J, Longy M, Sobol H and Birnbaum D (1997) Loss of heterozygosity in human breast carcinomas in the ataxia telangiectasia, Cowden disease and *BRCA1* gene regions. *Oncogene* **14**: 339–347

Kong D, Suzuki A, Zou TT, Sakurada A, Kemp LW, Wakatsuki S, Yokoyama T, Yamakawa H, Furukawa T, Sato M, Ohuchi N, Sato S, Yin J, Wang S, Abraham JM, Souza RF, Smolinski KN, Meltzer SJ and Horii A (1997) *PTEN1* is frequently mutated in primary endometrial carcinomas (letter). *Nat Genet* **17**: 143–144

Lancaster JM, Wooster R, Mangion J, Phelan CM, Cochran C, Gumbs C, Seal S, Barfoot R, Collins N, Bignell G, Patel S, Hamoudi R, Larsson C, Wiseman RW, Berchuck A, Iglehart JD, Marks JR, Ashworth A, Stratton MR and Futreal PA (1996) *BRCA2* mutations in primary breast and ovarian cancers. *Nat Genet* **13**: 238–240

Li DM and Sun H (1997) TEP1, encoded by a candidate tumor suppressor locus, is a novel protein tyrosine phosphatase regulated by transforming growth factor beta. *Cancer Res* **57**: 2124–2129

Li J, Yen C, Liaw D, Podsypanina K, Bose S, Wang SI, Puc J, Miliaresis C, Rodgers L, McCombie R, Bigner SH, Giovanella BC, Ittmann M, Tycko B, Hibshoosh H, Wigler MH and Parsons R (1997) *PTEN*, a putative protein tyrosine phosphatase gene mutated in human brain, breast, and prostate cancer (see comments). *Science* **275**: 1943–1947

Liaw D, Marsh DJ, Li J, Dahia PL, Wang SI, Zheng Z, Bose S, Call KM, Tsou HC, Peacocke M, Eng C and Parsons R (1997) Germline mutations of the *PTEN* gene in Cowden disease, an inherited breast and thyroid cancer syndrome. *Nat Genet* **16**: 64–67

Miki Y, Swensen J, Shattuck-Eidens D, Futreal PA, Harshman K, Tavtigian S, Liu Q, Cochran C, Bennett LM, Ding W, Bell R, Rosenthal J, Hussey C, Tran T, McClure M, Frye C, Hattler T, Phelps R, Haugen-Strano A, Katcher H, Yakumo K, Gholami Z, Shaffer D, Stone S, Bayer S, Wray C, Bogden R, Dayananth P, Ward J, Tonin P, Narod S, Bristow PK, Norris FH, Helvering L, Morrison P, Rosteck P, Lai M, Barrett C, Lewis C, Neuhausen S, Cannon-Albright L, Goldgar D, Wiseman R, Kamb A and Skolnick MH (1994) A strong candidate for the breast and ovarian cancer susceptibility gene *BRCA1*. *Science* **266**: 66–71

Miki Y, Katagiri T, Kasumi F, Yoshimoto T and Nakamura Y (1996) Mutation analysis in the *BRCA2* gene in primary breast cancers. *Nat Genet* **13**: 245–247

Nelen MR, van Staveren WC, Peeters EA, Hassel MB, Gorlin RJ, Hamm H, Lindboe CF, Fryns JP, Sijmons RH, Woods DG, Mariman EC, Padberg GW and Kremer H (1997) Germline mutations in the *PTEN/MMAC1* gene in patients with Cowden disease. *Hum Mol Genet* **6**: 1383–1387

Rasheed BK, Stenzel TT, McLendon RE, Parsons R, Friedman AH, Friedman HS, Bigner DD and Bigner SH (1997) *PTEN* gene mutations are seen in high-grade but not in low-grade gliomas. *Cancer Res* **57**: 4187–4190

Remmele W, Hildebrand U, Hienz HA, Klein PJ, Vierbuchen M, Behnken LJ, Heicke B and Scheidt E (1986) Comparative histological, histochemical, immunohistochemical and biochemical studies on oestrogen receptors, lectin receptors, and Barr bodies in human breast cancer. *Virchows Arch A Pathol Anat Histopathol* **409**: 127–147

Rhei E, Kang L, Bogomolniy F, Federici MG, Borgen PI and Boyd J (1997) Mutation analysis of the putative tumor suppressor gene *PTEN/MMAC1* in primary breast carcinomas. *Cancer Res* **57**: 3657–3659

Schmutzler RK, Bierhoff E, Werkhausen T, Fimmers R, Speiser P, Kubista E, Krebs D, Zeillinger R, Wiestler OD and Von Deimling A (1997) Genomic deletions in the *BRCA1*, *BRCA2* and *TP53* regions associate with low expression of the estrogen receptor in sporadic breast carcinoma. *Int J Cancer* **74**: 322–325

Singh B, Ittmann MM and Krolewski JJ (1998) Sporadic breast cancers exhibit loss of heterozygosity on chromosome segment 10q23 close to the Cowden disease locus. *Genes Chromosomes Cancer* **21**: 166–171

Sourvinos G, Kiaris H, Tsikkinis A, Vassilaros S and Spandidos DA (1997) Microsatellite instability and loss of heterozygosity in primary breast tumours. *Tumour Biol* **18**: 157–166

Spiessl B, Beahrs OB and Hermanek P (1990) *UICC TNM Atlas. Illustrated Guide to the TNM/pTNM Classification of Malignant Tumors*. Springer Verlag: Berlin

Steck PA, Pershouse MA, Jasser SA, Yung WK, Lin H, Ligon AH, Langford LA, Baumgard ML, Hattier T, Davis T, Frye C, Hu R, Swedlund B, Teng DH and Tavtigian SV (1997) Identification of a candidate tumour suppressor gene, *MMAC1*, at chromosome 10q23.3 that is mutated in multiple advanced cancers. *Nat Genet* **15**: 356–362

Tashiro H, Blazes MS, Wu R, Cho KR, Bose S, Wang SI, Li J, Parsons R and Ellenson LH (1997) Mutations in *PTEN* are frequent in endometrial carcinoma but rare in other common gynecological malignancies. *Cancer Res* **57**: 3935–3940

Tsou HC, Teng DH, Ping XL, Brancolini V, Davis T, Hu R, Xie XX, Gruener AC, Schrager CA, Christiano AM, Eng C, Steck P, Ott J, Tavtigian SV and Peacocke M (1997) The role of *MMAC1* mutations in early-onset breast cancer: causative in association with Cowden syndrome and excluded in *BRCA1*-negative cases (in process citation). *Am J Hum Genet* **61**: 1036–1043

Ueda K, Nishijima M, Inui H, Watatani M, Yayoi E, Okamura J, Yasutomi M, Nakamura Y and Miyoshi Y (1998) Infrequent mutations in the *PTEN/MMAC1* gene among primary breast cancers. *Jpn J Cancer Res* **89**: 17–21

von Deimling A, Bender B, Louis DN and Wiestler OD (1993) A rapid and non-radioactive PCR based assay for the detection of allelic loss in human gliomas. *Neuropathol Appl Neurobiol* **19**: 524–529

Wang SI, Puc J, Li J, Bruce JN, Cairns P, Sidransky D and Parsons R (1997) Somatic mutations of *PTEN* in glioblastoma multiforme. *Cancer Res* **57**: 4183–4186

Wooster R, Bignell G, Lancaster J, Swift S, Seal S, Mangion J, Collins N, Gregory S, Gumbs C and Micklem G (1995) Identification of the breast cancer susceptibility gene *BRCA2* (see comments) [published erratum appears in *Nature* (1996) **379**: 749]. *Nature* **378**: 789–792

World Health Organization (1981) *Histological Typing of Breast Tumours. 2nd International Classification of Tumors*. World Health Organization: Geneva

 © Cancer Research Campaign 1999
